# Trends in breast cancer mortality in Mexican women

**DOI:** 10.61622/rbgo/2025rbgo90

**Published:** 2025-11-18

**Authors:** Beatriz Villegas-Lara, Oswaldo Sinoe Medina-Gómez

**Affiliations:** 1 Mexican Institute of Social Security Clinical Epidemiology Research Unit Mexico City Mexico Clinical Epidemiology Research Unit, HGR 1, Mexican Institute of Social Security, Mexico City, Mexico.

**Keywords:** Breast neoplasms, Mortality, Spatial analysis, Health inequities, Mexico

## Abstract

**Objective:**

To estimate age-adjusted mortality rates at the national and state levels, evaluates trends and their spatial distribution during the period 2000-2023 among Mexican women.

**Methods:**

Ecological study conducted from open access data, the age-standardized mortality rate of breast cancer during the period 2000-2023 was calculated. Joinpoint regression models were estimated for breast cancer mortality in Mexico and its states. Spatial analysis was performed using Moran's I statistic.

**Results:**

The age-adjusted mortality rate at the national level during 2000 was 18.99 per 100,000 women, the highest mortality rate occurred in the year 2020. Joinpoint analysis shows a decrease in mortality in the last five years, being greater in urban areas. For 2023, the highest mortality occurred in Sonora and Chihuahua, while Mexico City (-0.71; 95%CI −0.98 to −0.44) was the entity that showed a significant reduction in the mortality rate from breast cancer. The spatial analysis showed a local indicator of spatial association of 0.458 (p<0.05) for 2000 and 0.524 (p<0.05) for 2023 in the northern states of the country.

**Conclusion:**

Public health interventions must be implemented according to the social, economic, and cultural context to reduce mortality from breast cancer.

## Introduction

Breast cancer (BC) represents a significant public health issue globally. Male breast cancer (MBC) accounts for approximately 1% of all breast cancer diagnoses. From 1990 to 2021, the incidence and mortality rate of MBC increased worldwide, with notable geographic disparities. However, these rates are projected to decrease by 2050.^(1,2)^ Breast cancer remains more prevalent among women. In 2022, an estimated 2,296,840 female breast cancer (FBC) cases were registered, with the highest incidence occurring in low- and middle-income countries. In North America, the incidence rate was 95.1 per 100,000 women, while in Latin America and the Caribbean, the age-adjusted incidence rate was 52.0 per 100,000 women.^(3)^

A relationship between the incidence of FBC and its survival has been identified with genetic predispositions such as BRCA mutations, hormonal processes, and reproductive patterns, however, there are other non-biological conditions such as access to information, educational level, socioeconomic level, and unemployment that are associated with breast cancer.^(4–6)^ Breast cancer is one of the leading causes of death in women worldwide. The age-adjusted mortality rate of FBC globally in 2022 was 12.7 per 100,000 women, with the highest mortality occurring in African countries (19.2 per 100,000 women). In the Americas, the highest mortality occurs in Uruguay, Argentina, and Paraguay.^(7)^ According to GLOBOCAN 2022, BC is the second leading cause of cancer deaths in Mexican women with an age-adjusted mortality rate of 12.2 per 100,000 women,^(7)^ which is why various screening programs have been implemented for the timely detection of BC in Mexico, including the promotion of self-examination, Clinical examination and annual or biannual mammography for women aged 40 to 49 years with risk factors and once a year for all women aged 50 years and older to reduce deaths from this cause.^(8)^

Despite the above, there are no recent studies that analyze trends in mortality from FBC in Mexico. Therefore, the objective of this study was to estimate age-adjusted mortality rates at the national and state levels, evaluate trends and their spatial distribution during the period 2000-2023 among Mexican women.

## Methods

An ecological epidemiological study was carried out where all records of deaths occurring from January 1, 2000, through December 31, 2023, were included in women aged 30 to 79 years whose basic cause of death was registered under code C50 (breast cancer) of the International Classification of Diseases and Related Health Problems, tenth edition (ICD-10).

The data were collected directly from the database published by the National Institute of Geography and Statistics.^(9)^ They were imported into the SPSS program, and the variables were labeled according to the data dictionary issued by the same institute. All records of deaths occurring in the period 2000 to 2023 in women aged 30 to 79 years whose basic cause of death was record under code C50 (breast cancer) of the International Classification of Diseases and Related Health Problems, tenth edition (ICD-10) were included. Deaths devoid of information regarding sex, age and place of habitual residence of the deceased were excluded.

Crude breast cancer mortality rates were estimated at the national and state levels according to the number of deaths divided by the number of people each year according to the registry reported by the National Population Council (CONAPO).^(10)^ Subsequently, the adjustment of mortality rates was made by the direct method, considering as the reference population women aged 30 to 79 years that reported for the country in 2023.

The analysis of adjusted mortality rate trends at the national level, by state of habitual residence, and by population type (rural vs. urban) was conducted using the Joinpoint Regression Program, version 5.0.2. The model assumed constant variance and no autocorrelation, with a logarithmic transformation applied to the data. This method allowed us to fit the data and identify temporal trends through joinpoint models. Based on the magnitude and direction of changes in mortality trends due to BC, the model identified statistically significant inflection points and provided estimates of the annual percentage change (APC), average annual percentage change (AAPC), and their 95% confidence intervals for the study period.^(11)^

Each attachment point reported a change in trend. A maximum of 4 binding points and a p-value <0.05 were statistically significant. The final model selected was the most parsimonious model that the program identified according to Bayesian information criteria.

GeoDA software was used to evaluate the spatial distribution. Moran's I statistic was used, whose values range approximately from 1 (positive spatial autocorrelation, perfect grouping of rates) to −1 (negative spatial autocorrelation, spatial dispersion). A reference distribution was used using 999 random permutations to indicate statistical significance with a value of p<0.05.

The Local Spatial Association Indicator (LISA) estimated through the local bivariate Moran statistical analysis, made it possible to identify whether mortality rates in the years 2000 and 2023 were clustered, dispersed or random considering the adjusted mortality rate for breast cancer in women aged 30 to 79 years. The result allowed us to recognize six types of spatial clusters:

1. Low-Low: Spatial units with low values significantly surrounded by units that also have low values with respect to the variable of interest.
2. Low-High: Presence of units with low values in the variable significantly surrounded by neighbors with high values.
3. High-Low: Presence of units with high values in the variable under study significantly surrounded by neighbors with low values.
4. High-High: Presence of units with high values in the variable under study significantly surrounded by neighbors with high values.
5. Non-significant relationship: Presence of units where the value of the variable of interest is not significantly related to the values presented by their neighbors.

The study was approved by the local ethics and research committee 3609 of the Mexican Institute of Social Security with registration number R-2024-3609-070.

## Results

**Figure 1:**
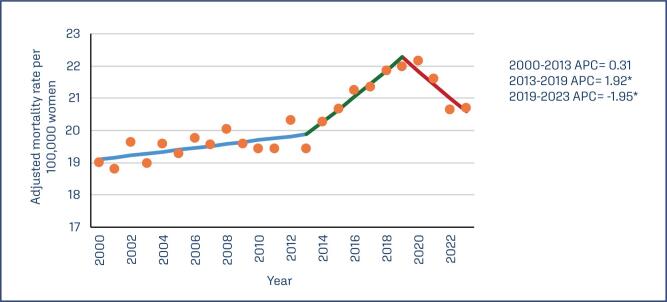
Annual percent change in breast cancer mortality in Mexican women, 2000-2023

During the period studied, 119,081 deaths from breast cancer occurred in women aged 30 to 79 years. The age-adjusted mortality rate at the national level during 2000 was 18.99 per 100,000 women, while the highest mortality rate occurred in 2020 (22.15 per 100,000 women) (Figure 1).

APC: Annual Percentage Change; *p< 0.05

**Source:** prepared by the authors based on the results.

The highest mortality from BC at the state level during 2000 occurred in Nuevo León (28.9 per 100,000 women), followed by Mexico City (28.0 per 100,000 women), while by 2023 the highest mortality occurred in Sonora (31.8 per 100,000 women) and Chihuahua (29.0 per 100,000 women) (Table 1).

**Table 1:** Age-adjusted mortality rate* for breast cancer in women aged 30 to 79 years by state of residence in Mexico

State of residence	2000	2001	2002	2003	2004	2005	2006	2007	2008	2009	2010	2011	2012	2013	2014	2015	2016	2017	2018	2019	2020	2021	2022	2023
Aguascalientes	20.2	18.5	20.9	20.5	22.2	23.3	17.9	21.6	30.1	22.6	17.4	22.0	24.8	21.3	23.0	21.2	24.4	26.8	30.4	22.6	23.9	19.1	19.2	21.7
Baja California	24.3	24.6	22.1	22.7	23.2	25.0	24.3	27.3	25.3	26.3	25.9	26.0	25.4	26.2	26.5	28.4	23.3	23.6	32.0	29.6	28.7	28.1	24.8	23.5
Baja California Sur	27.4	27.3	25.0	39.2	27.6	28.0	37.0	35.5	31.4	27.4	25.3	31.2	25.8	21.6	23.8	17.2	18.3	33.2	39.4	26.6	30.4	29.4	28.2	23.6
Campeche	8.9	10.4	13.3	10.3	14.0	14.6	22.1	8.2	13.4	16.6	14.1	12.3	12.5	13.1	9.3	12.6	17.2	8.7	17.7	19.0	16.9	19.0	15.9	10.4
Coahuila	24.1	27.3	25.3	27.2	23.5	22.3	24.3	29.7	23.6	25.9	25.4	26.8	28.8	29.4	24.7	29.9	29.7	29.9	25.7	27.8	30.6	26.4	26.0	27.2
Colima	18.4	20.0	34.6	19.8	20.4	21.6	26.7	27.5	20.1	23.7	27.4	23.6	28.2	24.3	28.1	24.7	27.7	29.1	15.6	19.8	30.0	32.5	25.5	25.7
Chiapas	8.7	8.0	10.0	11.4	10.6	10.2	11.2	10.5	13.7	13.3	14.8	15.4	15.8	15.6	16.6	13.9	17.5	14.1	14.8	17.1	16.9	16.8	15.4	13.2
Chihuahua	25.6	23.2	26.4	27.3	27.1	25.6	27.0	25.9	31.0	30.6	26.6	30.4	25.4	23.4	35.6	31.3	31.9	30.8	30.1	29.0	30.3	26.7	28.2	29.0
Ciudad de México	28.0	26.2	25.9	26.1	24.0	25.9	24.2	24.4	25.2	24.4	23.7	23.7	23.1	21.7	22.7	23.3	23.2	22.5	23.9	23.5	23.6	22.6	24.4	22.2
Durango	17.4	16.9	15.2	15.7	15.5	18.2	21.0	22.5	19.6	14.2	16.5	18.8	22.4	17.5	19.8	22.0	23.9	23.5	22.2	27.0	28.9	23.7	24.1	21.1
Guanajuato	14.6	16.1	20.4	15.7	21.5	17.1	20.3	18.7	17.8	17.7	16.1	19.8	19.8	22.0	20.9	20.7	22.1	21.8	22.6	25.7	23.0	22.9	21.6	20.9
Guerrero	10.4	11.2	10.5	12.2	13.8	11.6	13.2	12.4	13.1	13.3	14.6	12.2	12.5	12.0	13.7	13.6	15.0	15.1	16.4	16.6	14.3	15.2	14.1	13.6
Hidalgo	15.1	14.4	12.8	15.2	14.3	16.3	13.9	12.6	12.3	15.0	11.7	16.4	15.6	14.1	13.3	15.5	14.2	15.5	15.6	18.0	17.2	14.9	17.2	16.0
Jalisco	23.9	21.4	25.7	23.8	24.2	26.7	27.6	22.3	24.1	22.1	23.7	26.7	26.5	26.7	25.8	26.1	27.5	27.2	27.2	24.1	26.0	26.2	23.4	26.5
México	16.3	19.1	19.7	18.7	20.2	19.0	19.0	18.2	19.9	18.0	19.0	18.4	18.5	16.5	18.2	18.8	18.9	19.9	18.7	19.9	20.3	19.4	18.6	18.7
Michoacán	17.2	16.1	17.4	14.3	15.3	15.1	18.1	18.2	19.2	21.6	18.8	18.0	18.9	15.7	18.9	18.7	20.4	16.5	20.4	19.4	18.5	20.0	19.8	20.4
Morelos	11.7	13.9	16.6	19.4	19.1	17.3	16.6	20.0	19.8	15.1	19.2	19.9	21.6	17.3	13.7	19.4	16.4	21.0	14.8	20.9	16.2	19.4	19.3	21.1
Nayarit	20.1	14.4	18.5	20.6	19.5	17.4	19.6	17.1	16.6	19.9	14.4	16.2	16.7	15.9	20.6	15.7	21.7	22.1	28.9	20.4	19.7	19.8	23.8	20.3
Nuevo León	28.9	24.7	28.7	21.9	24.9	25.4	25.5	26.8	30.2	26.7	26.4	24.1	26.1	26.4	29.5	28.7	27.0	27.7	26.4	26.1	29.0	27.3	28.4	26.7
Oaxaca	10.5	9.9	10.0	12.3	11.3	10.8	11.5	11.2	12.4	7.8	9.7	8.2	9.2	9.6	11.3	12.1	11.4	13.1	13.9	13.1	14.6	15.9	14.4	15.7
Puebla	12.4	14.2	14.4	13.6	13.7	12.3	13.7	17.0	14.8	15.7	13.8	13.0	15.9	16.6	15.6	14.4	16.2	16.7	18.1	16.7	19.6	17.2	17.0	18.0
Querétaro	15.1	17.8	14.0	18.4	23.9	23.1	24.1	18.7	16.5	24.6	20.2	18.0	22.5	21.7	24.7	18.0	20.6	25.1	24.7	21.2	21.7	22.7	19.8	24.2
Quintana Roo	11.1	10.7	17.6	10.4	7.9	7.3	11.4	14.7	11.5	12.1	12.5	14.2	14.8	17.4	15.6	21.9	18.3	19.5	14.4	21.0	15.3	20.2	18.4	14.8
San Luis Potosí	12.5	16.2	13.6	11.6	13.2	13.8	17.3	18.1	18.6	18.2	18.6	18.5	17.5	13.5	17.9	17.0	21.2	20.4	18.0	21.6	23.5	23.5	21.1	20.0
Sinaloa	19.7	16.1	19.6	19.9	21.3	22.3	22.3	19.1	18.5	23.5	19.9	16.6	24.7	22.9	19.9	24.3	27.6	24.7	25.5	23.5	23.9	23.1	20.6	22.8
Sonora	26.1	24.3	22.8	23.0	28.0	25.6	21.6	22.8	30.6	23.0	28.3	22.5	27.3	26.1	27.0	33.3	27.4	28.8	27.4	28.2	29.6	27.9	26.6	31.8
Tabasco	13.3	19.1	14.5	12.0	15.8	16.9	13.5	11.6	16.8	16.3	17.9	15.4	17.1	16.5	15.5	19.0	16.9	17.9	19.6	22.4	21.7	20.9	18.8	20.6
Tamaulipas	22.4	24.1	25.6	22.4	25.3	24.7	25.7	24.7	20.1	24.3	25.4	23.0	22.2	25.2	23.5	25.2	27.1	25.6	27.5	25.4	26.4	29.0	23.9	26.0
Tlaxcala	8.2	12.8	16.6	14.4	13.0	12.8	13.3	16.6	15.3	15.9	12.5	13.0	14.9	16.2	14.8	14.1	14.8	19.9	18.9	16.2	18.4	13.2	15.4	15.6
Veracruz	15.6	14.5	15.8	15.9	16.1	16.0	16.7	17.0	16.0	17.0	16.8	16.4	18.7	17.1	16.8	16.5	18.2	19.1	19.6	21.8	20.1	21.0	18.7	18.7
Yucatán	15.2	11.7	11.3	11.7	17.2	9.4	9.8	10.1	7.6	14.0	11.9	15.2	14.4	15.4	15.9	15.7	16.4	15.5	14.1	14.3	14.9	14.7	14.0	15.0
Zacatecas	16.8	17.3	12.9	20.2	15.3	15.7	13.6	21.0	19.9	14.8	17.1	15.4	22.1	23.1	20.9	22.3	21.2	17.0	23.4	19.7	20.1	20.6	20.1	20.1

* per 100,000 women

**Source:** Compiled by the author based on the findings.

The joinpoint analysis indicates a slight increase in mortality rates between 2000 and 2013, followed by a greater increase in breast cancer mortality from 2013 to 2019 (APC = 1.92; p = 0.003), and a subsequent decrease during 2019-2023 (APC = −1.95; p = 0.009). In urban areas, there was a reduction in breast cancer mortality from 2000 to 2013 (APC = −0.25; p = 0.010); however, an increase in mortality was observed from 2013 to 2020 (APC = 1.01; p < 0.001), with a significant decrease from 2020 to 2023 (APC = −2.27; p < 0.001). In rural areas, there was a significant increase in mortality between 2000 and 2004 (APC = 5.90; p < 0.001), followed by a non-significant stabilization of mortality from 2004 to 2023 (APC = 0.54; p = 0.25) (Figure 1).

**Figure 2:**
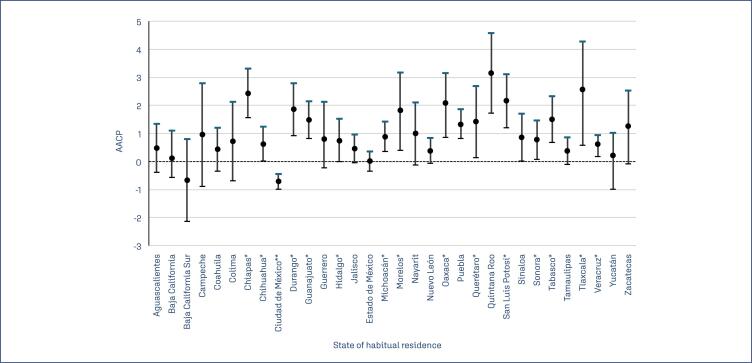
Average annual percentage change in breast cancer mortality by state of habitual residence in Mexico

The states with the highest AAPC are Quintana Roo (2.88; 95%CI: 1.45 to 4.31), Tlaxcala (2.69; 95% CI: 0.63 to 4.44), and Chiapas (2.61; 95% CI: 1.74 to 3.49), while Mexico City (-0.60; 95%CI −0.87 to −0.33) showed a significant reduction in breast cancer mortality rate (Figure 2).

AAPC: Average Annual Percentage Change

*p value <0.005

**Source:** prepared by the author based on the results.

The APC trends (Table 2) across states were heterogeneous. In most states, no significant changes were observed during the study period. However, some entities showed distinct patterns: for example, in Morelos, breast cancer mortality increased during the initial segment of the study, followed by a stable period. In Yucatán, mortality declined between 2000 and 2008 but increased again from 2008 to 2011 (APC: 18.81; 95% CI: 4.18–26.19).

**Table 2:** Annual percent change in breast cancer mortality by state of habitual residence in Mexico

State	Period	APC	95%CI
Aguascalientes	2000-2023	0.48	-0.38 to 1.35
Baja California	2000-2021	1.06	0.55 to 4.34
	2021-2023	-9.31	-16.44 to 0.53
Baja California Sur	2000-2023	-0.67	-2.13 to 0.81
Campeche	2000-2023	0.97	-0.88 to 2.79
Coahuila	2000-2023	0.47	-0.35 to 1.21
Colima	2000-2023	0.77	-0.68 to 2.13
Chiapas	2000-2012	5.39	3.75 to 8.36
	2012-2023	-0.69	-3.61 to 1.06
Chihuahua	2000-2023	0.62	0.01 to 1.24
Mexico City	2000-2013	-1.39	-2.60 to −0.95
	2013-2023	0.17	-0.48 to 2.39
Durango	2000-2023	1.87	0.93 to 2.80
Guanajuato	2000-2023	1.48	0.82 to 2.15
Guerrero	2000-2019	1.72	0.92 to 11.57
	2019-2023	-3.39	-16.44 to 1.30
Hidalgo	2000-2023	0.75	-0.01 to 1.52
Jalisco	2000-2023	0.46	-0.05 to 0.96
México	2000-2023	0.01	-0.34 to 0.36
Michoacán	2000-2023	0.89	0.36 to 1.43
Morelos	2000-2003	14.99	2.17 to 41.19
	2003-2023	-0.02	-2.44 to 0.82
Nayarit	2000-2023	1.01	-0.13 to 2.10
Nuevo Len	2000-2023	0.38	-0.07 to 0.84
Oaxaca	2000-2007	1.68	-2.17 to 14.68
	2007-2010	-9.48	-14.09 to 12.32
	2010-2023	5.17	-3.89 to 8.98
Puebla	2000-2023	1.33	0.82 to 1.86
Querétaro	2000-2023	1.43	0.13 to 2.70
Quintana Roo	2000-2023	3.16	1.73 to 4.59
San Luis Potosí	2000-2023	2.17	1.21 to 3.12
Sinaloa	2000-2023	0.86	0.01 to 1.71
Sonora	2000-2023	0.78	0.07 to 1.47
Tabasco	2000-2023	1.5	0.68 to 2.32
Tamaulipas	2000-2023	0.38	-0.10 to 0.86
Tlaxcala	2000-2002	27.21	1.86 to 56.00
	2002-2023	0.49	-3.87 to 1.39
Veracruz	2000-2015	0.7	-0.56 to 1.14
	2015-2019	4.86	1.90 to 8.21
	2019-2023	-3.66	-8.90 to −1.22
Yucatán	2000-2008	-5.32	-11.09 to −2.47
	2008-2011	18.81	4.18 to 26.19
	2011-2023	-0.25	-6.71 to 1.61
Zacatecas	2000-2023	1.25	-0.09 to 2.54

APC: Annual Percentage Change; 95%CI: 95% confidence interval

**Source:** prepared by the author based on the results.

**Figure 3:**
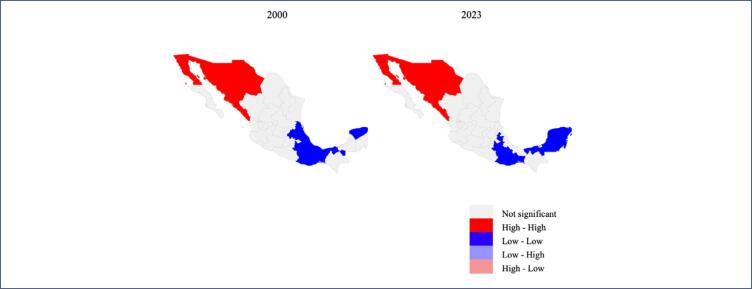
Breast cancer mortality clusters in Mexican women

The value of Moran's I for the year 2000 was 0.458 (p<0.005) while in 2023 it was 0.524 (p<0.005). Figure 3 shows a significant spatial grouping on the map according to age-adjusted breast cancer mortality rates for the years 2000 and 2023. A statistically significant group was detected for the occurrence of "High-High" (red) in the northwestern states of the country made up of the states of Baja California, Sonora, Chihuahua and Sinaloa, while the "Low-Low" group (blue) was identified in 6 states in the center of the country (Puebla, Oaxaca, Tabasco, Campeche, Quintana Roo and Yucatán), which had low rates. also surrounded by provinces with lower rates than the average.

## Discussion

This study shows trends in breast cancer mortality in Mexico over the past 23 years. The results show that there were three phases where initially the moderate increase in mortality occurred in the first 13 years and later in a period of 6 years there is a high mortality and subsequently, the APC decreases in an inverse proportional way to the previous period.

Breast cancer is a condition that has a significant impact on women's lives, modifying various aspects of their lives.^(12)^ Although significant efforts have been made to reduce mortality from BC (breast cancer), there is still a significant percentage of women who have not undergone self-examination, undergone a mammogram, or have obtained adequate mammographic screening.^(13)^

The trend of breast cancer mortality among women is consistent with patterns observed in 29 countries with very high human development indices (HDI), which contrasts with the increasing rates found in countries with low HDI. In our study, the annual percentage change (APC) is −1.95, indicating a decline similar to those seen in Argentina, Chile, and Uruguay.^(12)^ This reduction surpasses the global average, where the estimated annual percentage change (EAPC) in deaths decreased by 0.56% per year.^(14–17)^

An upward trend in mortality from BC has been reported in urban areas compared to rural areas.^(18,19)^ However, in our study, the trend in mortality in urban areas showed a downward trend in the last three years, which can be explained by the age adjustment made for the analysis of this study, considering that older women live in urban areas. In addition, in rural areas there is a lack of specialists in rural areas, specialized health centers for comprehensive cancer care, and people have lower incomes that allow them to access private care if they do not have care in public services.^(19,20)^

This study shows trends in breast cancer mortality in Mexico over the past 23 years. The results show that there were three phases where initially the moderate increase in mortality occurred in the first 13 years and later in a period of 6 years there is a high mortality and subsequently, the APC decreases in an inverse proportional way to the previous period.

The study shows the heterogeneity in breast cancer trends among states, which may be a result of the efficiency of cancer prevention and treatment actions at the local level and allows us to identify possible needs for the implementation of specific actions for the control and timely treatment of breast cancer.^(21)^

On the other hand, changes in APC of breast cancer mortality among states over 23 years may indicate the degree of efficacy in cancer prevention and treatment depending on the area and suggest the need for area-specific applications of different cancer control programs.^(21)^

The geographical distribution of areas with the highest breast cancer mortality between 2000 and 2023 is mainly concentrated in the northern region of the country. This pattern overlaps with states characterized by high mortality rates and a low percentage of the population lacking access to health services. The concentration in northern states may be partly explained by increased genetic susceptibility, particularly the G119T mutation in the CYP1B1 variant of cytochrome P450, which is associated with estrogen metabolism.^(22)^

That is why the spatial analysis of conditions such as breast cancer is essential to identify high- priority strata or areas to identify barriers in the availability of information for the general population, access and availability of care, training of general practitioners and gynecologists of first contact and treatment.^(23–25)^

Previous studies have shown a positive relationship with breast cancer mortality and lower socioeconomic status.^(26,27)^ However, in our study, this relationship was not identified in the geographic clusters, although it is necessary to recognize that a limitation was that the socioeconomic status of women who died from breast cancer was not analyzed at the individual level, and possibly in entities with low mortality from breast cancer there may be a low notification of events derived from barriers in diagnosis. That is why the recognition of these geographic concentrates would make it possible to identify in subsequent studies the equitable distribution of infrastructure and supplies necessary for the screening and timely care of breast cancer, which play an important role in the differences found in trends in breast cancer mortality at the national and state levels.^(28,29)^

Finally, differences in health care and health policies, including those geared towards early detection and treatment, as well as sociocultural and environmental factors, could play an important role in the differences found in trends and geographic distribution of breast cancer mortality and implement targeted multilevel interventions at the individual level, community and regional.^(30,31)^

Therefore, these types of studies based on trends and socio-spatial distribution are useful tools to potentially identify additional local conditions or needs to address female breast cancer in these specific sites.^(32)^

Derived from the type of study carried out, the possibility of the ecological fallacy must be considered without detracting from the results obtained. The type of analysis does not allow us to identify changes in trends due to the implementation of strategies for promotion, prevention and access to medical care.

## Conclusion

The present study shows that in recent years there has been a decrease in breast cancer mortality in women in Mexico. At the state level, trends are mixed, with some states showing significant increases in recent years. In addition, geographic areas where breast cancer mortality is concentrated were identified. It is imperative to analyze the impact of detection and treatment strategies, among others, that have specifically influenced the changes in observed trends. Public health interventions should be tailored to the social, economic, and cultural context to reduce breast cancer mortality. It is important to enhance efforts for the early detection of breast cancer, offer timely and personalized treatment based on genetic susceptibility, and remove barriers to accessing health services.
